# Multiple Locus Variable-Number Tandem-Repeat and Single-Nucleotide Polymorphism-Based *Brucella* Typing Reveals Multiple Lineages in *Brucella melitensis* Currently Endemic in China

**DOI:** 10.3389/fvets.2017.00215

**Published:** 2017-12-14

**Authors:** Mingjun Sun, Zhigang Jing, Dongdong Di, Hao Yan, Zhicheng Zhang, Quangang Xu, Xiyue Zhang, Xun Wang, Bo Ni, Xiangxiang Sun, Chengxu Yan, Zhen Yang, Lili Tian, Jinping Li, Weixing Fan

**Affiliations:** ^1^Laboratory of Zoonoses, Chinese Animal Health and Epidemiology Center, Qingdao, China; ^2^Xinjiang Center of Animal Disease Control, Urumqi, China; ^3^Laboratory of Exotic Disease, Chinese Animal Health and Epidemiology Center, Qingdao, China; ^4^Department of Animal Disease Epidemiological Investigation, Animal Health and Epidemiology Center, Qingdao, China

**Keywords:** *Brucella melitensis*, MLVA, multilocus sequence typing, whole-genome sequence, single-nucleotide polymorphism, phylogeny

## Abstract

Brucellosis is a worldwide zoonotic disease caused by *Brucella* spp. In China, brucellosis is recognized as a reemerging disease mainly caused by *Brucella melitensis* specie. To better understand the currently endemic *B. melitensis* strains in China, three *Brucella* genotyping methods were applied to 110 *B. melitensis* strains obtained in past several years. By MLVA genotyping, five MLVA-8 genotypes were identified, among which genotypes 42 (1-5-3-13-2-2-3-2) was recognized as the predominant genotype, while genotype 63 (1-5-3-13-2-3-3-2) and a novel genotype of 1-5-3-13-2-4-3-2 were second frequently observed. MLVA-16 discerned a total of 57 MLVA-16 genotypes among these *Brucella* strains, with 41 genotypes being firstly detected and the other 16 genotypes being previously reported. By BruMLSA21 typing, six sequence types (STs) were identified, among them ST8 is the most frequently seen in China while the other five STs were firstly detected and designated as ST137, ST138, ST139, ST140, and ST141 by international multilocus sequence typing database. Whole-genome sequence (WGS)-single-nucleotide polymorphism (SNP)-based typing and phylogenetic analysis resolved Chinese *B. melitensis* strains into five clusters, reflecting the existence of multiple lineages among these Chinese *B. melitensis* strains. In phylogeny, Chinese lineages are more closely related to strains collected from East Mediterranean and Middle East countries, such as Turkey, Kuwait, and Iraq. In the next few years, MLVA typing will certainly remain an important epidemiological tool for *Brucella* infection analysis, as it displays a high discriminatory ability and achieves result largely in agreement with WGS-SNP-based typing. However, WGS-SNP-based typing is found to be the most powerful and reliable method in discerning *Brucella* strains and will be popular used in the future.

## Introduction

Brucellosis is one of the world’s most important zoonotic diseases causing great damage to husbandry industry and public health (1). While some developed countries have successfully eradicated this disease, it remains the major concern for many developing countries in Africa, South America, and much of Asia including China (2–9). Brucellosis is caused by intracellular Gram-negative bacteria belonging to *Brucella* genus. So far six species have been officially identified based on differences in pathogenicity, host preferences, and conventional phenotyping methods: *Brucella abortus* (cattle), *Brucella melitensis* (goat and sheep), *Brucella suis* (pigs), *Brucella canis* (dogs), *Brucella ovis* (sheep), and *Brucella neotomae* (rodents). More recently, additional species have been suggested originating from a wide range of hosts, *Brucella ceti* (cetaceans), *Brucella pinnipedialis* (seals), *Brucella microti* (voles), *Brucella papionis* (baboons), and *Brucella vulpis* (foxes) (10–12).

In China, brucellosis is regarded as a remerging disease with previous high incidence occurred in 1950s–1980s (13, 14). In 1960s, a comprehensive control measures including animal vaccination, test-slaughter, and movement restriction were introduced to control this disease, thereafter a low-incidence period was observed from 1980s to 1990s. During that period, brucellosis incidence in domestic animals was kept at below 0.3%, while human brucellosis rate ranged around 0.05–0.10 cases per 100,000 residents. However, during the past decades, outbreaks of brucellosis in domestic animals and human have been increasingly reported, especially in north of China where brucellosis was highly endemic in history, such as Inner Mongolia, Xinjiang, Qinghai, and Ningxia. In recent years, brucellosis is rapidly spreading across the mainland with the trend from northern area to southern provinces where brucellosis was not historically serious. Over a long period of time, test-slaughter as major measure has been implemented to control brucellosis in domestic animals, now this strategy is proven to be less effective. To alleviate the worsening *Brucella* infection, vaccination on domestic animals was reintroduced to Inner Mongolia in 2012. Until 2015, the overall outbreaks of brucellosis in cattle and sheep had been significantly decreased (from 4,741 in 2012 to 1,534 in 2015), although it remained as the most severe brucellosis-endemic region according to data shown in Chinese Official Veterinary Bulletin.^1^ Contemporarily, other provinces with no vaccination implemented saw an increased number of brucellosis outbreaks in domestic animals, among them Xinjiang was mostly affected (from 413 in 2012 to 927 in 2015). According to the lately issued National Brucellosis Control Plan of China (2016–2020), a comprehensive of measures are going to be applied to control animal brucellosis. From 2016, all the provinces in northern China will carry out vaccination program against brucellosis in bovine and small ruminants, meanwhile more stringent rules will come into effect to restrict animal movement from brucellosis high-incidence region to low-incidence region. In support of the plan, bacteriological surveillance in domestic animal reservoir is specifically emphasized.

The knowledge on current major *Brucella* species, biovar and genotype, and their geographic distribution is of great valuable, especially for selecting an appropriate vaccine, tracking-back infections sources, and monitoring transmission routes. In China, a range of *Brucella* species and biovars has been reported in animals, including *B. melitensis* (biovar 1, 2, and 3), *B. abortus* (biovar 1–7, 9), *B. suis* (biovar 1 and 3), *B. canis*, and *B. ovis*. However, human brucellosis was mainly caused by *B. melitensis*, implying a major source from infected small ruminants. National sentinel surveillance has been established to monitor the seroprevalence of brucellosis in animals, but systematic bacteriological survey was seldom carried out. From 2009, authorized by Ministry of Agriculture of China (MoA), Chinese Animal Health and Epidemiology Center carried out etiological investigation on cattle and small ruminant brucellosis in Northern China where animal vaccination has not been conducted, such as Xinjiang, Shanxi, and Hebei provinces. So far, a quite number of *Brucella* isolates has been obtained and most of them were *B. melitensis*.

Here, in this study, for improving our understanding of the currently endemic *B. melitensis* strains in China, a popularly used *Brucella* typing method of MLVA-16 [multiple locus variable-number tandem-repeat (VNTR) assay] was applied to these *B. melitensis* strains collected by our lab. This *Brucella* typing scheme, utilizing 16 VNTRs, has been proven to have the ability to differentiate *Brucella* species, biovar, and even the isolates (15, 16). More importantly, there is an online database of MLVA-16 profiles available to the all researchers allowing comparison of *Brucella* strains in the global scope.^2^ Another *Brucella* typing method based on whole-genome sequence (WGS) extracted single-nucleotide polymorphisms (SNPs) was also applied to these Chinese *B. melitensis* strains. It provides unprecedented resolution in deciphering phylogenetic relationships among different *Brucella* species (17, 18), as well as a great power to distinguish closely related isolates within a species (15, 19–24). As NGS technique becomes more affordable to many laboratories, full genome sequences of *Brucella* isolates with a diverse of geographical backgrounds can be available on publically accessible database. It provides a substantial foundation for widespread use of this reliable method in *Brucella* isolate typing and phylogenetic relationship analysis. In addition, the previously used multilocus sequence typing (MLST) based on smaller number of SNPs was also evaluated in this paper.

## Materials and Methods

### Strains Background

From 2010 to 2016, systematic bacteriological isolations were conducted on tissue and milk samples collected from cattle and small ruminants serologically positive to *Brucella* infection. A total of 110 *B. melitensis* strains were obtained from a multiple locations involving 7 Northern provinces and 50 counties. By further biotype characterization based on conventional serotyping, most of these *B. melitensis* strains were identified as biovar 3 (*n* = 99, 90%), and only a small part of strains were found to be biovar 1 (*n* = 9, 8%) and biovar 2 (*n* = 2, 2%).

### MLVA Genotyping

MLVA including eight minisatellite loci (panel 1: Bruce06, 08, 11, 12, 42, 43, 45, and 55) and eight microsatellite loci (panel 2, subdivided into panel 2A: Bruce18, 19, 21; and panel 2B: Bruce04, 07, 09, 16, and 30) was performed as previously described (15, 24). For each locus, Hunter and Gaston diversity index (HGDI) were calculated by online software.^3^ Cluster analysis on strains was conducted using BioNumerics software (version 7.6, Applied Maths, Belgium) and based on the categorical coefficient and UPGMA. Web-based MLVA database (see text footnote 2) was utilized for the convenience of comparing strains from different countries.

### MLST Typing

Multilocus sequence typing genotyping was performed with the method described by Whatmore et al. (25). To increase the discriminatory ability on *Brucella* strains, the more informative scheme including 21 loci (BruMLSA21) was used. Each new allele of the 21 loci was given a distinct numerical designation following up the PubMLST databases.^4^ Each unique allelic profile for these 21 loci was identified as a sequence type (ST). The assembled sequences of the 21 loci were then concatenated, and phylogenetic analyses on all identified STs were conducted using MEGA software (version 5.1) as described earlier.

### Whole-Genome Sequencing, Assembling, and Annotation

Whole-genome sequencing was performed on the Illumina HiSeq2000 platform at Novogene (Novogene, Beijing, China). The generated reads were assembled into contiges using *Brucella* 16M as the reference (GeneBank: NC003317 and GenBank: NC003318). GeneMarkS was used to retrieve the related coding gene. Transfer RNA (tRNA) genes were predicted by the tRNAscan-SE. Putative tRNA and rRNA genes were analyzed by the tRNAscan-SE and rRNAmmer, respectively. Small nuclear RNAs were predicted by BLAST against the Rfam database. Six databases were routinely used to predict gene functions including GO, KEGG, COG, NR, Swiss-Prot, and TrEMBL.

### WGS-SNP Discovery and Phylogenetic Tree Construction

We introduced Prokka to fully annotate draft bacteria genomes. All annotated assemblies in GFF3 format was used as input files for to conduct core-pan analysis (26). SNP sites were extracted from core gene alignment file generated by Roary (27). The missing and ambiguous data and gap were excluded. A matrix data containing the orthologous SNPs were generated. The filtered dataset was applied to conduct evolutionary analyses using the MEGA software (version 5.1). Neighbor-joining tree was constructed using Jukes–Cantor model and the percentage bootstrap confidence levels of internal branches were calculated from 1,000 resamplings of the original data. The *B. abortus* 2308 strain was selected as outgroup.

## Results

### MLVA Typing and Analysis

#### MLVA-8 Analysis

**Figure 1:**
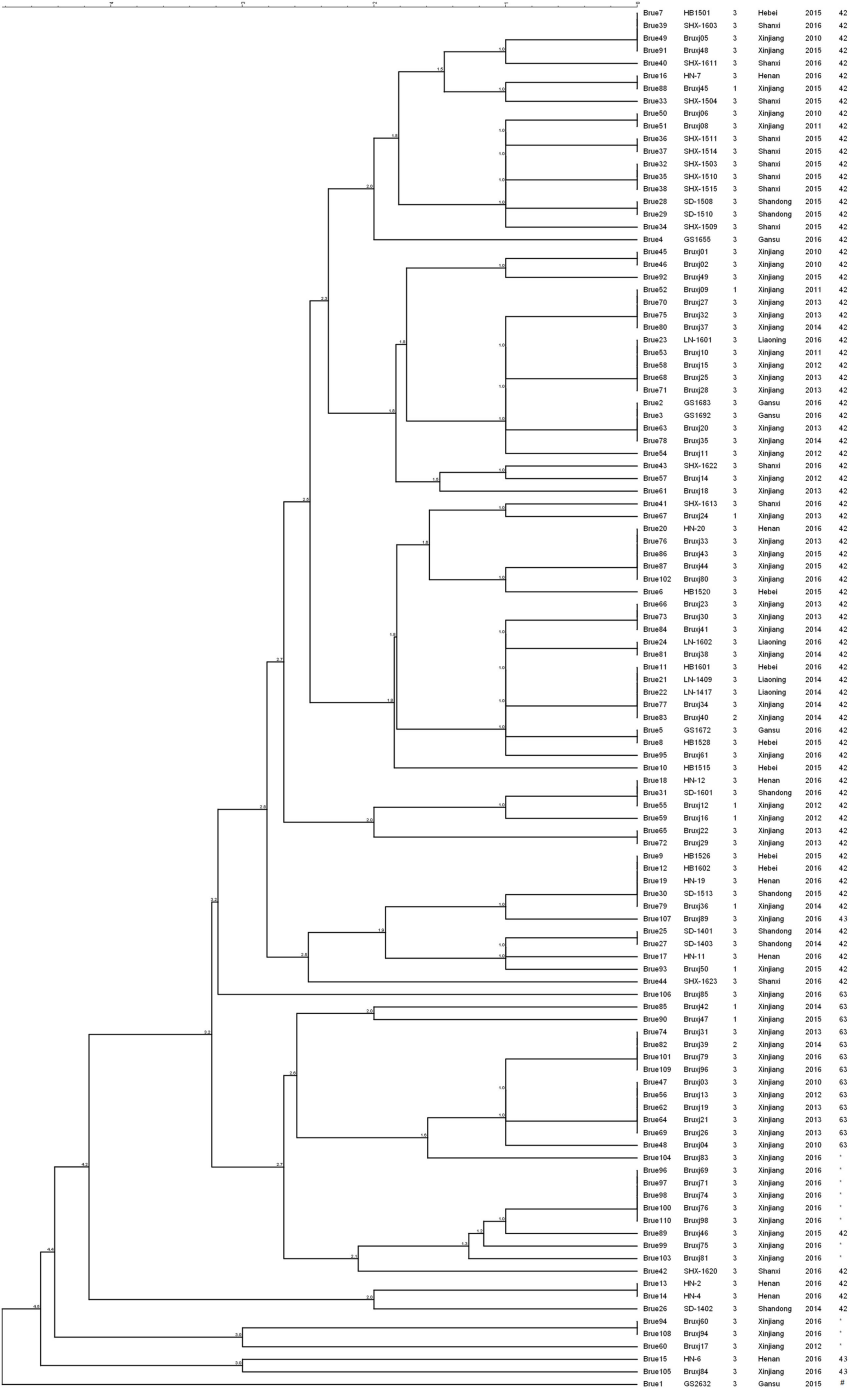
Clustering analysis of 110 *Brucella melitensis* isolated from China based on dataset of 16 variable-number tandem-repeats. In the columns, the following data are indicated: key, strain ID, biovar, isolation location, isolation date, and MLVA-8 genotype ID. * indicates the MLVA-8 genotype with the profile of 1-5-3-13-2-4-3-2; ^#^ indicates the newly detected MLVA-8 genotype of 1-5-3-14-2-2-3-2 in this study.

Among 110 *B. melitensis* strains, MLVA-8 typing comprising eight panel 1 loci generated five genotypes, with VNTR patterns described as 1-5-3-13-2-2-3-2 (82 strains, 75%), 1-5-3-13-2-3-3-2 (13 strains, 12%), 1-5-3-13-2-4-3-2 (11 strains, 10%), 1-5-3-13-3-2-3-2 (3 strains, 3%), and 1-5-3-14-2-2-3-2 (1 strain) (Table 1). Of them, three genotypes, previously numbered as genotype 42 (1-5-3-13-2-2-3-2), 63 (1-5-3-13-2-3-3-2), and 43 (1-5-3-13-3-2-3-2), are typical East Mediterranean lineage and have been reported in China (2, 16, 28–30). Genotype 42 is most frequently observed and widely distributed throughout the mainland, while genotype 63 and 43 are less reported and only endemic in local area such as Xinjiang, Inner Mongolia, Qinghai, and Shanxi. The genotype with the profile of 1-5-3-13-2-4-3-2 was the most lately identified and only detected previously in single *B. melitensis* strain collected from Xinjiang (31). Here, in this study, more strains were identified as the same MLVA-8 genotype, and they were exclusively collected from Xinjiang, suggesting the existence of a new lineage in this local area. The remaining MLVA-8 genotype (1-5-3-14-2-2-3-2) was never reported before. This novel profile may represent a highly mutated *B. melitensis* strain, as it is well separated from all others common strains according to MLVA-16 clustering tree (Figure 1).

**Table 1:** MLVA-8 genotypes identified in 110 Chinese *Brucella melitensis* strains.

MLVA-8 genotype	Copy no. in each variable-number tandem-repeat locus	No. of strains	Percentage
42	1-5-3-13-2-2-3-2	82	75
63	1-5-3-13-2-3-3-2	13	12
N/A	1-5-3-13-2-4-3-2	11	10
43	1-5-3-13-3-2-3-2	3	2
N/A	1-5-3-14-2-2-3-2	1	

Total		110	

#### MLVA-16 Analysis

MLVA-16 (including panel 1 and panel 2 loci) displayed higher discriminatory power and generated 57 genotypes within the same collection of *B. melitensis* strains (Figure 1). The high diversity revealed by MlVA-16 is mainly contributed by three panel 2B loci (Bruce04, 16, 30) with the HGDI values ranging from 0.743 to 0.777 (Table 2). By comparing with the data deposited in MLVA database, 41 genotypes comprising 70 strains were found to be unique to China, while the remaining 16 genotypes have been reported in Turkey and Kazakhstan (4, 8). In China, these 57 MlVA-16 genotypes demonstrate different geographic distribution. 47 genotypes are found to be provincially specific and the other 10 genotypes are shared by two or more provinces. MLVA-16 genotype described as 1-5-3-13-2-2-3-2-4-41-8-7-4-3-6-6 is most widely distributed genotype and shared by four provinces of Xinjiang, Hebei, Henan, and Shandong. Based on the diverse MLVA-16 genotypes identified in this study, especially those endemic in local regions, a proper tracking-back analysis for infection sources could be achieved.

**Table 2:** Number of alleles and HGDI values of *Brucella melitensis* strains isolated in China.

Variable-number tandem-repeat (VNTR) locus		Alleles number	Copy number of VNTR	HGDI	Confidence interval	Max (Pi)
Panel 1	Bruce06	1	1	0.000	0.000–0.064	1.000
	Bruce08	1	5	0.000	0.000–0.064	1.000
	Bruce11	1	3	0.000	0.000–0.064	1.000
	Bruce12	2	13, 14	0.018	0.000–0.053	0.991
	Bruce42	2	2, 3	0.071	0.006–0.136	0.964
	Bruce43	3	2, 3, 4	0.392	0.290–0.494	0.764
	Bruce45	1	3	0.000	0.000–0.064	1.000
	Bruce55	1	2	0.000	0.000–0.064	1.000
Panel 2A	Bruce18	2	4, 5	0.018	0.000–0.053	0.991
	Bruce19	2	41, 46	0.018	0.000–0.053	0.991
	Bruce21	1	8	0.000	0.000–0.064	1.000
Panel 2B	Bruce04	6	3, 4, 5, 6, 7, 8	0.743	0.702–0.783	0.382
	Bruce07	2	4, 5	0.054	0.000–0.111	0.973
	Bruce09	4	3, 7, 8, 9	0.106	0.027–0.184	0.945
	Bruce16	11	2, 3, 4, 5, 6, 7, 8, 9, 10, 12, 13	0.777	0.733–0.822	0.318
	Bruce30	8	4, 5, 6, 7, 8, 9, 10, 11	0.738	0.679–0.797	0.436

### MLST Typing

**Figure 2:**
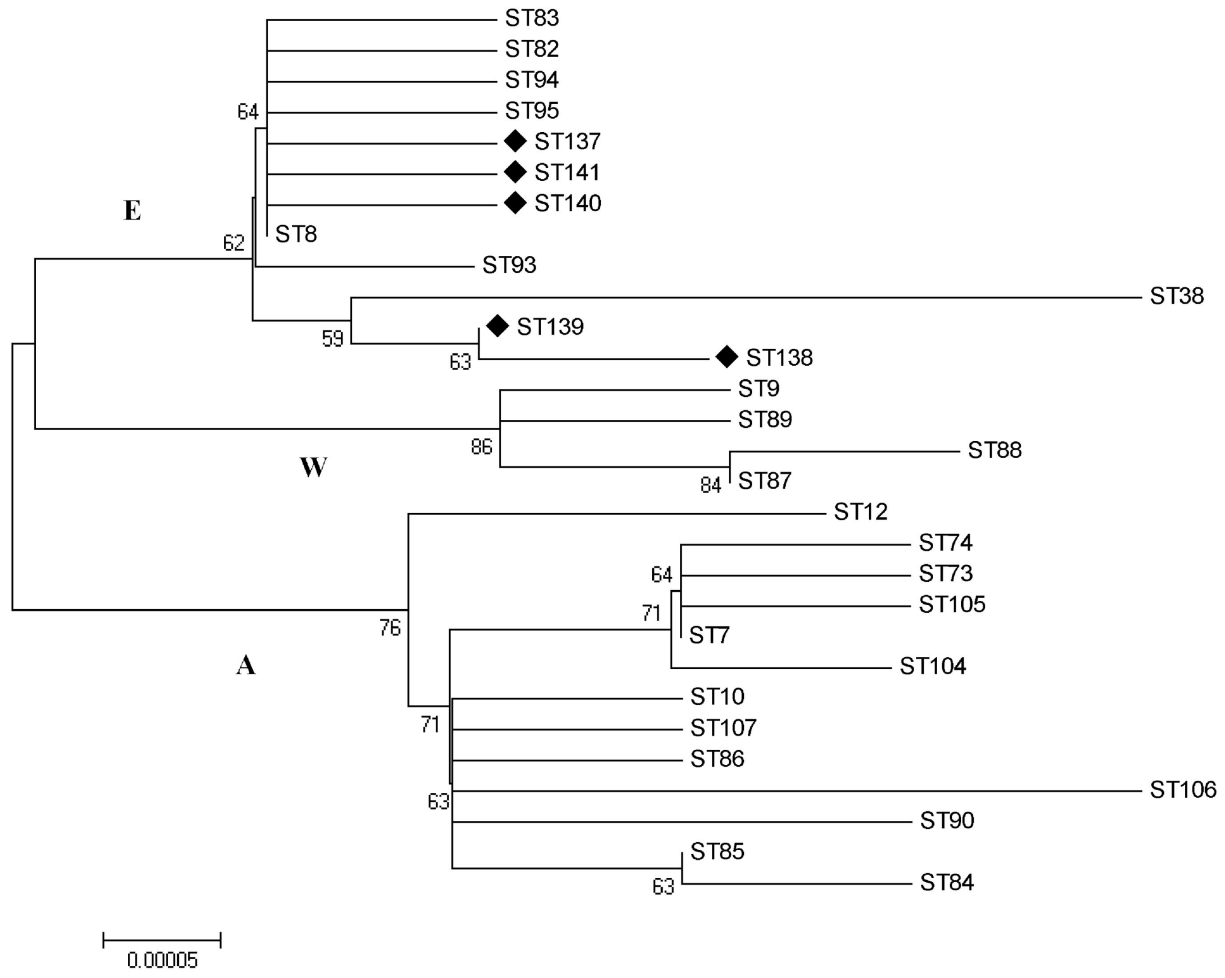
Phylogenetic analysis of newly detected ST137, ST138, ST139, ST140 and ST141 together with 24 sequence types (STs) previously reported in *Brucella melitensis*. In the phylogenetic tree, distinct three clades were observed, with A standing for America/Africa lineage, W and E for West Mediterranean and East Mediterranean lineages.

By BruMLSA21 typing, 6 STs were identified among these 110 Chinese *B. melitensis* strains (Table S1 in Supplementary Material). One ST, known as ST8, comprises 91 strains and is found to be predominant over all other STs. The other five STs were firstly detected and designated as ST137, ST138, ST139, ST140, and ST141 in MLST database (see Text footnote 4). ST139 consists of 15 strains obtained from three provinces of Shandong, Henan, and Xinjiang, representing the second frequently observed *B. melitensis* lineage in China. ST137, ST138, ST140, and ST141 were identified in individual strain. Phylogenetic tree was constructed using all *B. melitensis* STs deposited in database, including the five newly detected STs in this study (Figure 2). 29 STs were separated into three lineages with distinct geographic origins, Americas (Africa), West Mediterranean, and East Mediterranean (25). All novel STs detected in this study together with the predominant ST8 were clustered into East Mediterranean lineage, indicating a close phylogenetic relationship of Chinese *B. melitensis* strains to those from East Mediterranean region. This result is in agreement with the finding based on MLVA analysis. According to database, ST8 is the most common ST identified in *B. melitensis* and shows a wide distribution covering many European and Asian Countries. By phylogeny, ST137, ST140, and ST141 represent stains emerging from the prevailing ST8, while ST138 and ST139 represent another branch that is genetically connected to stains discovered from Egypt, Syria, and Iraq.

### General Genomic Features

Nine *B. melitensis* strains representing different MLVA-8 genotypes were subject to whole-genome sequencing. The generated scaffold sequence covered 99.77% of complete genomic sequence when compared to reference strain 16M. G/C content was calculated as 57.24%. An average of 3,291 protein-encoding genes (±5) were predicted among these draft genomes. One copy of 5s, 16s, and 23s subunits in rRNA operon were detected in each strain, meanwhile 50 ± 1 copies of tRNA and 17 ± 2 copies of sRNA were predicted within these genomes (Table 3).

**Table 3:** Genomic features of nine *Brucella melitensis* strains isolated from China.

Strain ID	MLVA-8 genotype	Scaffold no.	Genome size (bp)	Gene number	Transfer RNA	sRNA	TRF	T4SS
Bruxj85	63	25	3,288,124	3,294	50	17	80	13
Bruxj20	42	24	3,286,504	3,287	49	16	81	13
HN06	43	24	3,286,987	3,296	51	17	88	13
Bruxj21	63	25	3,287,098	3,289	51	17	78	13
Bruxj71	N/A	25	3,287,527	3,293	51	15	82	13
Bruxj09	42	27	3,286,702	3,291	51	18	83	13
HB1526	42	27	3,287,364	3,285	51	18	78	13
Bruxj08	42	28	3,287,443	3,289	51	19	81	13
Bruxj38	42	27	3,288,458	3,293	51	16	82	13

### WGS-SNP-Based Phylogenetic Analysis

To better understand the evolutionary status of Chinese *B. melitensis* strains in a global context, WGS-SNP-based phylogenetic tree was constructed using 81 *B. melitensis* genomes deposited in NCBI genome bank and the nine genomes of sequenced in this study (Table S2 in Supplementary Material). *B. melitensis* biovar 1 strain 16M (NC_003317 and NC_003318.1) was chosen as reference for SNP calling. By Roary, 8,903 SNPs were extracted from 1,798 core genes. After filtering the sites lying in positions containing gaps and missing data, a total of 6,421 reliable SNPs were obtained and used for phylogenetic analysis.

**Figure 3:**
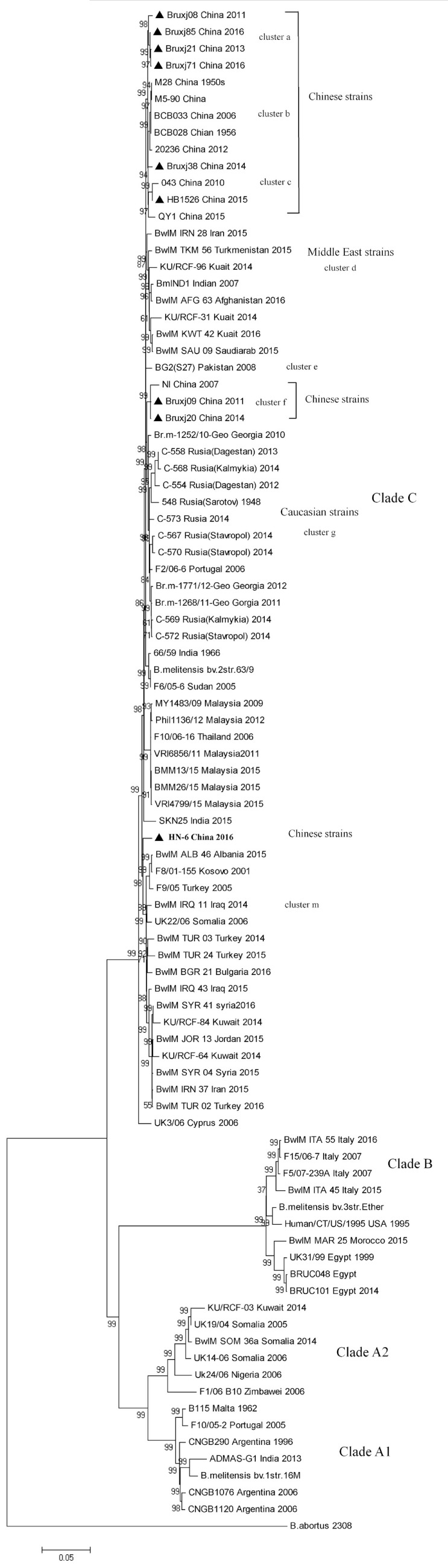
Whole-genome sequence-single-nucleotide polymorphism-based phylogenetic analysis of 9 *Brucella melitensis* strains isolated from China with the 81 *B. melitensis* strains selected from NCBI database. Neighbor-joining tree was constructed using Jukes–Cantor model and the setting of bootstraps was 1,000. *Brucella abortus* referential strain 2308 was included as outgroup. Strain information as ID, isolation location, and date are listed.

In phylogenetic tree, 90 *B. melitensis* strains with a diverse geographic backgrounds were clustered into four major clades (labeled as Clade A1, A2, B, and C) corresponding spatially to the potential origins of these strains (Figure 3). Clade A1 comprised strains mainly isolated from the Americas, while the clade A2 represented the strains with Africa sources. All strains collected from Italy, Egypt, and Morocco formed clade B, and in many literatures it was termed as Western Mediterranean lineage. The clade C, comprising the largest *B. melitensis* populations with a wide spatial distribution from the Mediterranean region to Asian countries, was recognized as Eastern Mediterranean lineage. 17 Chinese strains including the nine selected in this study were falling into Clade C, and further separated into five clusters (cluster a, b, c, f, and l). The cluster a, b, and c comprise the most strains unexceptionally collected from China (13/17), representing the main *B. melitensis* lineage uniquely endemic in China. These clusters emerge from the same internal node with cluster d comprising the strains collected from Middle East region, suggesting a common origin for *B. melitensis* endemic in these two regions (Figure 3). Cluster f (comprising NI, Bruxj09 and Bruxj20) represents a minor Chinese lineage and is closely related in phylogeny to the strains collected from Caucasian region, such as Georgia and some Russian Republics of Dagestan, Kalmykia, Sarotov, and Stavropol (cluster g). The strain HN-6 was clustered into cluster m, demonstrating a long distance in phylogenetic tree to other Chinese strains. It represents a rarely observed lineage in China, but frequently detected in East Mediterranean and Middle East countries such as Turkey, Syria, and Kuwait (cluster m). This result support previous findings based on MLVA analysis that Chinese *B. melitensis* lineages are fundamentally East Mediterranean region originated (2, 8, 31), but with a more definitive scope pointing to Middle East and Caucasian countries.

## Discussion

For many years, the traditional phenotyping based on host specificity, growth feature, biochemical reaction, serotyping, and bacteriophage typing has been the golden standard for *Brucella* characterization at both species and biovar levels. In China, *B. melitensis* has long been recognized as the main species causing brucellosis in human and domestic animals. However, at the subspecies level, change of predomiant biovar has been observed in past years. In previous high-incidence stage (1950s–1980s), biovar 1 was the most frequently observed and accounted for around 55% of *B. melitensis* strains collected from human patients and infected animals. While in current reemergence stage, biovar 3 is most often observed and responsible for 90% of small ruminant infections as data shown in this study. Studies on human brucellosis also revealed the similar result (28, 30). Existence of a predominant biotype in a brucellosis-endemic area makes the species and biovar as epidemiological markers much less significant, especially in tracing newly occurred cases to their sources. Thus, other *Brucella* typing methods with higher discriminatory power are particularly needed.

Of the *Brucella* typing methods used so far, MLVA has been proven to be highly powerful in discerning *Brucella* isolates and was popularly used over global laboratories. MLVA-8 genotyping (based on panel 1 loci) may provide a limited discriminatory power, but it is still informative for understanding the relationships between *Brucella* isolates with a diverse geographic sources. Before this paper, a decade MLVA-8 genotypes have been reported in China, of which genotypes 42 and 63 are the mostly frequently detected genotypes with a relatively wider distribution than other genotypes (2, 28–30, 32, 33). The most recently detected genotype (1-5-3-13-2-4-3-2) in this study represents the single-locus variants from already existed genotype 42 or 63, implying the ongoing mutation of *B. melitensis* in a specific niche. Further MLVA-16 genotyping (panel 1 and 2 loci) revealed much more diversity, which is mainly due to three highly variable loci (Bruce04, 16, and 30) and one loci with medium variability (Brue09) (Table 2). In these loci, tandem-repeat numbers show successive one-unit difference, implying a stepwise mutation mode taken by *B. melitensis* in a specific environment. It also suggests that the novel MLVA-16 genotypes identified in this study may represent the most recently mutated strains from a few ancestors. Up to now, around 120 MLVA-16 genotypes have been identified among *B. melitensis* strains collected in China, with which proper epidemiological investigations in outbreaks of human and animal brucellosis can be achieved.

**Figure 4:**
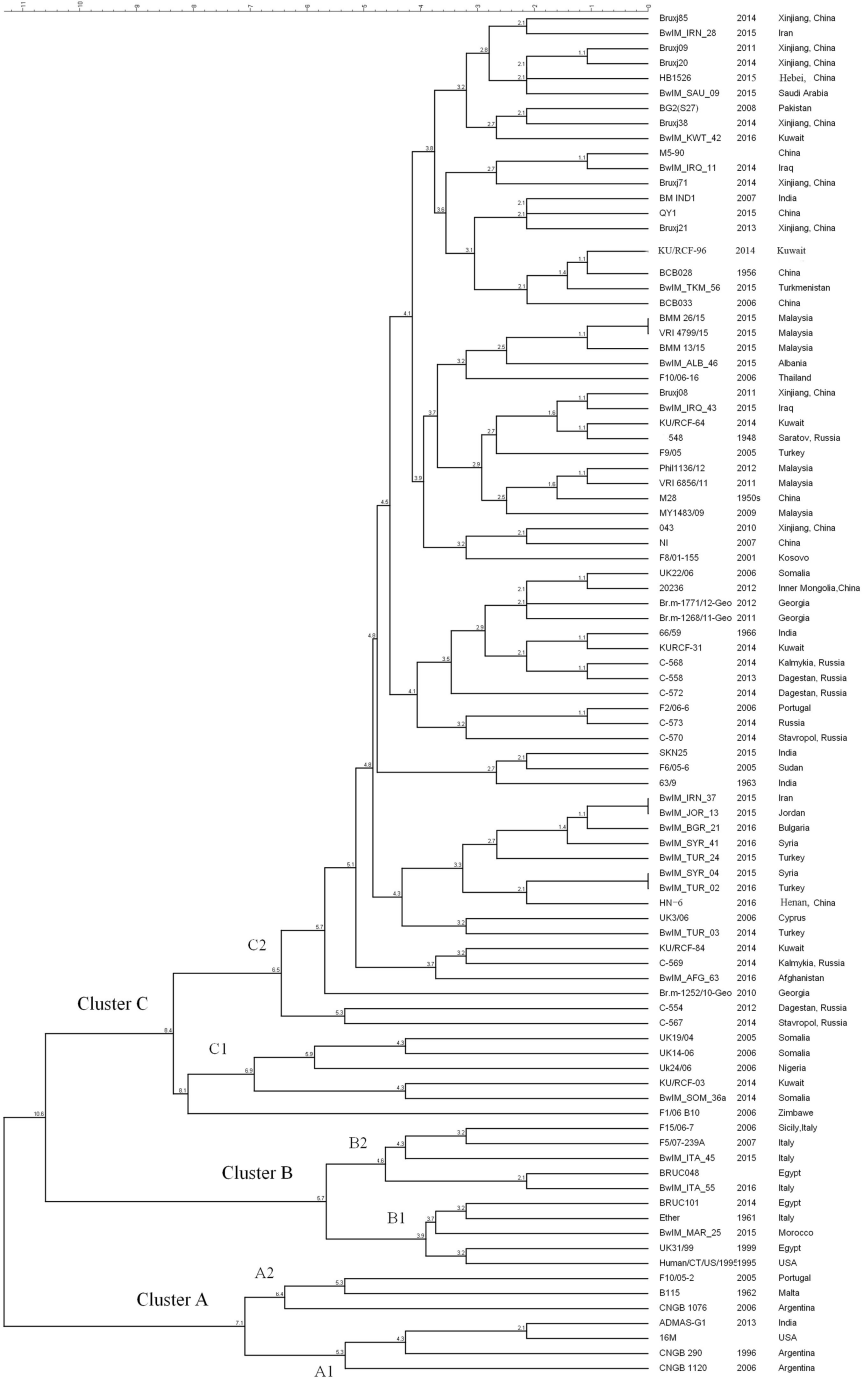
MLVA-16 clustering analysis of 90 *Brucella melitensis* strains including 81 strains selected from NCBI database and 9 strains used in this study.

As MLVA-16 is comparatively inexpensive and a large database is available, it will certainly remain an important epidemiological tool for *Brucella* infections analysis in the near future. The reliability of this method was partially confirmed by WGS-SNP-based phylogenetic analysis. As data shown in this paper, 90 *B. melitensis* strains with diverse geographic origins were separated into similar clusters in both WGS-SNP phylogenetic and MLVA-16 clustering trees (Figures 3 and 4). The Cluster A, B, C1, and C2 discerned by MLVA-16 analysis is perfectly corresponding in their respective to Clade A1, B, A2, and C in phylogenetic tree, making the strains from America, Africa, Western Mediterranean, and Eastern Mediterranean/Asia backgrounds clearly separated. However, WGS-SNP-based phylogeny demonstrated higher reliability than MLVA-16 clustering, given its ability in resolving strains from a narrower geographic region. For example, the Chinese strains (cluster a, b, and c), which were well grouped together in phylogenetic tree, scattered across MLVA-16 clustering tree and intersected with other strains. SNPs appear to be a better choice for *Brucella* typing and related phylogenetic analysis, as they are evolutionarily stable, and more importantly, thousands of SNPs scattered in genome provide the finest resolution. As NGS becomes more affordable to local laboratories, the widely accepted MLVA-16 will be inevitably replaced by more reliable SNPs typing based on WGSs.

Multilocus sequence typing has been also accepted as a tool for bacterial typing and epidemiological studies, and the resulting data is as well ideal for phylogenetic studies. The previous BruMLSA9 scheme, utilizing 9 discrete genomic loci comprising of 4,396 bp, detected around 30 SNPs from worldwide collected *B. melitensis* isolates and resolved them into 6 STs (from ST7 to ST12) (34). By this scheme, the majority of *B. melitensis* strains endemic in China were identified to be ST8, and a few novel STs were also detected (31, 35). The recently developed BruMLSA21 (comprising 10,244 bp within 21 gene loci) displayed higher discriminatory power and detected a total of 29 STs worldwide, including the 5 novel STs identified in this study. Comparing to MLVA-16 and WGS-SNP-based typing, BruMLSA21 provided a limited ability in discerning highly homogeneous *B. melitensis* isolates. However, with a robust MLST database available to all researchers, it remains useful for strain comparison.

## Conclusion

Due to the homogeneity of the *B. melitensis* species, especially under the current circumstance that the epidemic *Brucella* agents are overwhelmingly *B. melitensis* biovar 3, the traditional biotyping is of limited epidemiological value. WGS-SNP-based typing is so far the most powerful tool in differentiating *Brucella* isolates, with which multiple lineages were identified among *B. melitensis* strains currently circulating in China. In evolutionary relationship, the Chinese lineages are more closely connected to the strains from East Mediterranean and Middle East countries, such as Turkey, Kuwait, and Iraq. In the near future, MLVA typing will certainly remain an important epidemiological tool for *Brucella* infections analysis, as it displays a high discriminatory ability and achieves result largely in agreement with WGS-SNP-based typing. Based on the large number of MLVA-16 genotypes obtained so far, proper epidemiological investigations could be carried out when outbreaks of human and animal brucellosis occurred in China.

## Author Contributions

MS designed the study, analyzed the data, and drafted the manuscript. ZJ made WGS-SNP calling. DD, HY, ZZ, QX, and XZ undertook the data analysis work. XW, BN, XS, and CY performed portions of experiment. ZY, LT, and JL were responsible for strain collection and storage. WF oversaw the biosafety issue.

## Conflict of Interest Statement

The authors declare that the research was conducted in the absence of any commercial or financial relationships that could be construed as a potential conflict of interest.

## Footnotes

^1^http://www.moa.gov.cn/zwllm/tzgg/gb/sygb/.

^2^http://microbesgenotyping.i2bc.paris-saclay.fr.

^3^http://www.hpa-bioinformatics.org.uk/cgi-bin/DICI/DICI.pl.

^4^http://pubmlst.org/brucella/.

## Supplementary Material

Table S1 MLVA-16 dataset of 110 *Brucella melitensis* strains collected from 2010 to 2016 in China.

Table S2 MLVA-16 dataset of 90 *Brucella melitensis* used for whole-genome sequence-single-nucleotide polymorphism-based phylogenetic analysis.
